# Sulfoxide Reductases and Applications in Biocatalytic Preparation of Chiral Sulfoxides: A Mini-Review

**DOI:** 10.3389/fchem.2021.714899

**Published:** 2021-08-19

**Authors:** Tao Peng, Xiaoling Cheng, Yongzheng Chen, Jiawei Yang

**Affiliations:** ^1^ Department of Biochemistry, Zunyi Medical University, Zunyi, China; ^2^ Key Laboratory of Biocatalysis and Chiral Drug Synthesis of Guizhou Province, Zunyi Medical University, Zunyi, China

**Keywords:** methionine sulfoxide reductase, DMSO reductase, chiral sulfoxide, kinetic resolution, biocatalysis

## Abstract

Chiral sulfoxides are valuable organosulfur compounds that have been widely used in medicinal and organic synthesis. Biocatalytic approaches for preparing chiral sulfoxides were developed in the past few years, mainly through asymmetric oxidation of prochiral sulfides. Recently, the application of sulfoxide reductase to prepare chiral sulfoxides through kinetic resolution has emerged as a new method, exhibiting extraordinary catalytic properties. This article reviews the chemical and biological functions of these sulfoxide reductases and highlights their applications in chiral sulfoxide preparation.

## Introduction

Sulfoxide is a chemical compound containing a sulfinyl (SO) functional group attached to two carbon atoms. Chiral sulfoxides are noteworthy reagents in a wide range of industrial fields (Khiar, 2003; Han et al., 2018). Optically pure sulfoxides play significant roles in asymmetric synthesis, either as chiral building blocks or as stereodirecting groups (Han et al., 2018). Moreover, chiral active sulfoxides have been widely applied as pharmaceuticals (Goundry et al., 2017). They have been reported to have lots of pharmaceutical activities, including anti-bacterial, anti-fungal, anti-ulcer, anti-artherosclerotic, anti-helminthic, and antihypertensive (Olbe et al., 2003; Babiak et al., 2011; Goundry et al., 2017). Usually, only one of the enantiomers could show the desired bioactivity; hence, both academia and industry are highly interested in the enantioselective preparation of chiral sulfoxides.

Enantiomerically pure sulfoxides could be obtained using chemical or biological methods (Khiar, 2003; Han et al., 2018). In the last few years, biocatalysis has emerged as an effective method for preparing high added-value products (Kiełbasiński, 2011; Zheng and Xu, 2011; Matsui et al., 2014; Choi et al., 2015; Patel, 2018). The main advantages of biocatalytic methods are that enzymes can achieve high efficiency and enantioselectivity under mild reaction conditions. The general biocatalytic approaches for preparing chiral sulfoxides include asymmetric oxidation of sulfides and kinetic resolution of racemic (*rac*) sulfoxides. A great number of oxidases, including Baeyer–Villiger monooxygenases (de Gonzalo et al., 2010; Rioz-Martínez et al., 2010; Bisagni et al., 2013; Bordewick et al., 2018; Zhang et al., 2018; Liu et al., 2021), cytochrome P450 monooxygenases (Zhang et al., 2010; Wu et al., 2018), peroxidases (Sangar et al., 2012; Bassanini et al., 2017), and dioxygenases (Hibi et al., 2011; Shainsky et al., 2013), have been used for the synthesis of chiral sulfoxides. Meanwhile, biocatalytic kinetic resolution of *rac*-sulfoxides has also become an attractive approach for preparing chiral sulfoxides (Anselmi et al., 2021), although it was relatively less reported. In this approach, the undesired sulfoxide enantiomer is transformed, while the desired one remained unaltered and recovered. Three general kinetic resolution approaches have been reported, including asymmetrical reduction to sulfides (Anselmi et al., 2021), oxidiation to sulfones (Li et al., 2011), and modification in the side chains (Kiełbasiński et al., 2008).

Chemical or biological molecules containing the sulfoxide group also exist in living cells, playing important roles in cellular metabolism (McCrindle et al., 2005). In cells, there are sulfoxide reductases in charge of the reduction of these sulfoxides to the corresponding sulfides. Three kinds of sulfoxide reductases have been found, including methionine sulfoxide reductase (Msr) active on methionine sulfoxides (Brot and Weissbach, 2000), dimethylsulfoxide (DMSO) reductase active on DMSO (McCrindle et al., 2005), and biotin sulfoxide reductase active on biotin sulfoxide (Pollock and Barber, 2001; Pollock et al., 2003; Denkel et al., 2013). In general, the reductase is mainly active on one of the two enantiomers of sulfoxide substrate. The high enantioselectivity of these enzymes implies the potential in the kinetic resolution of *rac*-sulfoxides to prepare chiral sulfoxides. Till now, Msr and DMSO reductases have been reported to be used in chiral sulfoxide preparation. In particular, Msr shows extremely high substrate tolerance toward a series of aryl alkyl sulfoxides (Yang et al., 2019). In this mini-review, we provide an overview of these two kinds of sulfoxide reductases and their important applications in chiral sulfoxides preparation.

### Methionine Sulfoxide Reductase

L-methionine (L-Met) is a proteinogenic amino acid with a sulfide moiety. In living cells, methionine is relatively easily oxidized to sulfoxide (L-Met-O) under mild conditions (Stadtman et al., 2003; Achilli et al., 2015). For many proteins, oxidation of Met residues to the sulfoxide would result in loss of their biological properties (Stadtman et al., 2003). Moreover, the free L-Met-O can negatively regulate some biosynthetic processes, such as being unable to combine with adenosine to form S-adenosylmethionine (Achilli et al., 2015). In cells, the oxidation of Met residues is readily reversed through the methionine sulfoxide reductase (Msr) catalyzed reduction of Met-O back to Met (Drazic and Winter 2014). The first evidence of an enzymatic activity that catalyzed the reduction of Met-O was obtained in rats in the late 1930s (Bennett, 1937). The exact enzyme that reduces free Met-O was finally identified and purified from *E. coli* in 1980, named methionine sulfoxide reductase (Msr) (Ejiri et al., 1980). In addition, two other enzymes called thioredoxin (Trx) and thioredoxin reductase (TrxR) function as electrons transferred from NADPH to Msr during the reduction process (Russel and Model, 1986). It is believed that the cyclic interconversion between Met and Met-O in proteins is involved in lots of biological processes, including antioxidation, regulation of enzyme activities, cell signaling, and proteolytic degradation (Stadtman et al., 2003; Achilli et al., 2015).

**FIGURE 1:**
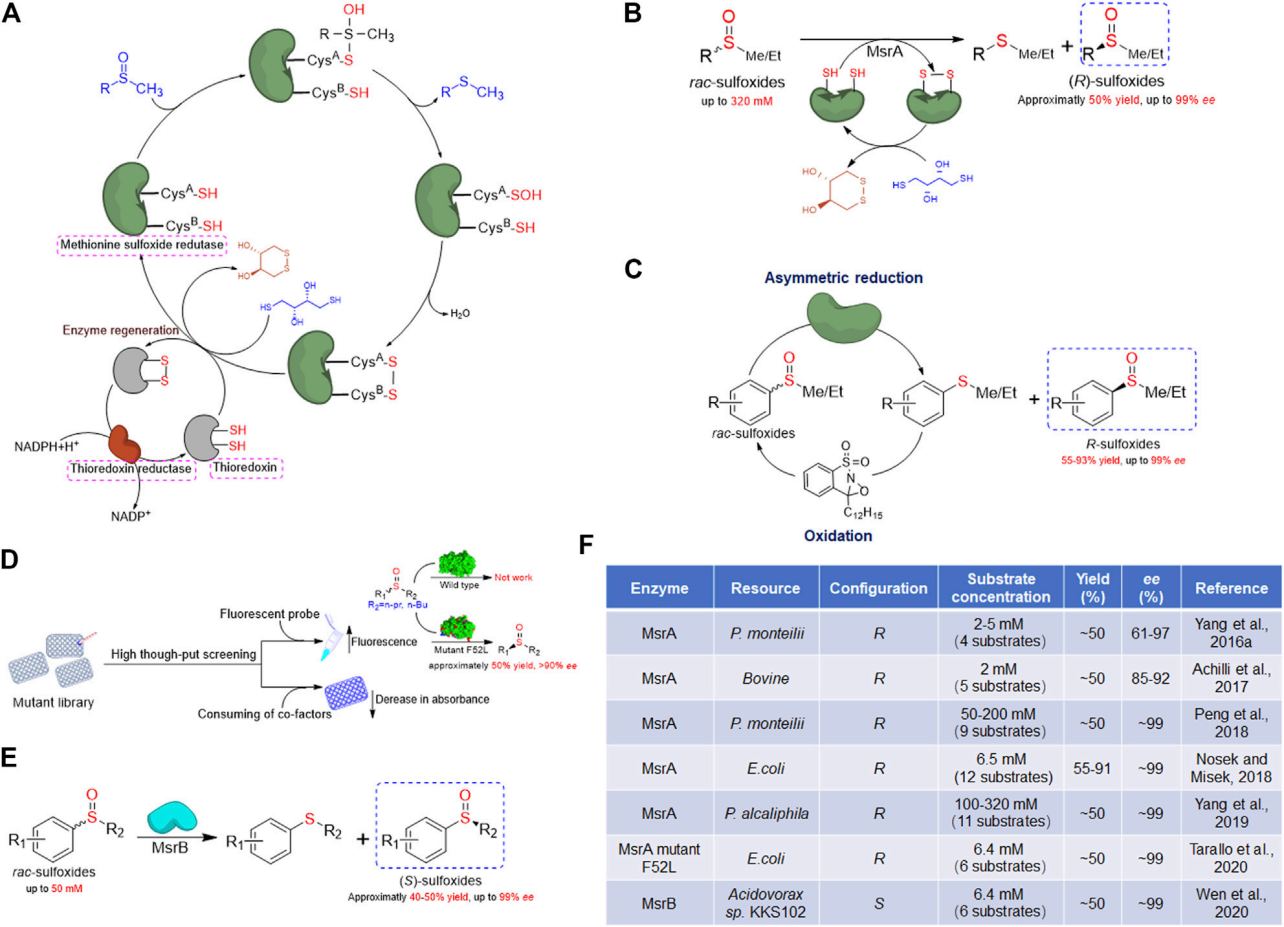
Overview of methionine sulfoxide reductase and application in chiral sulfoxides preparation. **(A)** General catalytic mechanism of methionine sulfoxide reductase. **(B)** Schematic presentation of kinetic resolution catalyzed by MsrA for (*R*)-sulfoxides preparation. **(C)** Schematic presentation of cascade chemoenzymatic reaction for (*R*)-sulfoxides preparation by MsrA and oxaziridine. **(D)** Schematic presentation of high-throughput screening strategies of MsrA and the latest achievement in modification of substrate scope. **(E)** Schematic presentation of kinetic resolution catalyzed by MsrB for (*S*)-sulfoxides preparation. **(F)** Summary of Msr enzymes applied for preparation of chiral sulfoxides through kinetic resolution.

Oxidation of Met creates a chiral center at the sulfur atom, making the produced Met-O a mixture of S and R enantiomers (Boschi-Muller et al., 2005). Therefore, different types of reductases are active on each enantiomer of Met-O due to their enantioselectivity. For instance, the presence of a total of six Msrs has been identified in *E. coli*. Three Msrs are active toward Met-*S*-O: MsrA that reduces Met-*S*-O in both free and protein-bound forms, MsrA1 that is only active on peptide-bound Met-*S*-O, and fSMsr that only reduces the free Met-*S*-O. Two forms reduce Met-*R*-O called MsrB and fRMsr, which are mainly active on the Met-*R*-O in protein-bound and free forms, respectively. The sixth enzyme called mem-*R*, *S*-Msr, a membrane-bound protein, catalyzes the reduction of both Met-O enantiomers, either free or inserted into peptides (Achilli et al., 2015). Msrs have been found in almost all organisms, from prokaryotes to eukaryotes (Boschi-Muller and Branlant, 2014; Achilli et al., 2015). To date, plenty of Msr structures have been solved, revealing that they are structurally unrelated (Drazic and Winter 2014; Tossounian et al., 2015; Kim, 2020; Kim et al., 2021). Nevertheless, these enzymes share a similar catalytic mechanism that involves one to three conserved cysteine residues (Drazic and Winter 2014). Most Msrs contain two cysteine residues (one catalytic cysteine and one recycling cysteine), but some Msrs have one catalytic cysteine and two recycling cysteines or only one catalytic cysteine. Despite these varieties, the catalytic cycle of Msr contains three common steps (Weissbach et al., 2002; Boschi-Muller et al., 2005). Typically, the first step is a nucleophilic attack to the Met-O substrate by the catalytic cysteine, resulting in the formation of a sulfenic acid intermediate with a concomitant release of corresponding Met. The recycling cysteine or reduced glutathione then attacks the sulfenic acid intermediate and forms an intra-monomeric disulfide bond with releasing one H_2_O molecule. This intra-monomeric disulfide bond is lastly reduced by thioredoxin, and the Msr is reactivated (Figure 1A). Dithiothreitol (DTT) could substitute the thioredoxin to break the intra-monomeric disulfide bond to reactivate the enzyme *in vitro* (Achilli et al., 2015). Due to the specific reduction that one kind of Msr is only active on one sulfoxide enantiomer, these enzymes provide an excellent solution for preparing chiral sulfoxides through kinetic resolution. To date, several MsrA and MsrB recombinant proteins from different species have been applied for chiral sulfoxide preparation.

### Application of MsrA in Chiral Sulfoxides Preparation

MsrA catalyzes the conversion of Met-*S*-O to reduced Met, working on both free and protein-bound forms. The first gene encoding MsrA was cloned and sequenced from *E. coli* in the 1990s (Rahman et al., 1992; Brot et al., 1995). Later in 1996, Moskovitz et al. reported the cloning, sequencing, and expression of the mammalian MsrA and showed that this enzyme reduced both natural and synthetic sulfoxide substrates (Moskovitz et al., 1996). To date, researches have shown the ubiquitous distribution of MsrA, from prokaryotes to eukaryotes. Most of MsrA contain a conserved motif consisting of amino acids Gly-Cys-Phe-Trp-Gly required for MsrA catalytic activity and a C-terminal thioredoxin binding domain (Kim and Gladyshev, 2007). Data from the 3D structures of the MsrA in complex with substrate show that the substrate-binding site is composed of a hydrophilic subsite, including Glu94, Tyr82, and Tyr134, which stabilizes the oxygen of the sulfoxide, and a hydrophobic subsite, including Trp53 and Phe52, which binds the ε methyl group of the sulfoxide (numbering based on the *E. coli* MsrA sequence) (Boschi-Muller and Branlant, 2014).

Due to the natural biochemical reactivity of MsrA that is specifically active on the Met*-S*-O, MsrA could be used as a biocatalyst to perform the kinetic resolution for the preparation of (*R*)-sulfoxides. Tremendous progress in the application of MsrA enzymes for preparing chiral sulfoxides through kinetic resolution has been achieved in the last 5 years. In 2016, our group reported the first application of MsrA for the preparation of (*R*)-sulfoxides (Yang et al., 2016a). Initially, a bacterial strain named *Pseudomonas monteilii* CCTCC M2013683 was isolated in our lab and exhibited the activity for the synthesis of chiral sulfoxides (Chen et al., 2014). After transcriptional analysis of this strain, a gene encoding MsrA (named *pm*MsrA) was noticed (Yang et al., 2016a). The whole-cell system expressing recombinant *pm*MsrA protein was then used as a biocatalyst to prepare (*R*)-sulfoxides through the reductive resolution of *rac*-sulfoxides. After optimizing the conditions, the phenyl methyl sulfoxide and several halogen substitutes on the aromatic ring were obtained with approximately 50% yield and more than 90% enantiomeric excess (*ee*) in the *R* configuration (Yang et al., 2016b). This finding provides new insight into the preparation of optically active sulfoxides. Later, Minetti et al. have expressed a recombinant mammalian MsrA and evaluated the enantiospecificity toward several aryl methyl sulfoxides, yielding the *R* enantiomer also with good optical purity (Achilli et al., 2017).

Previous reports have shown that higher substrate concentrations usually lead to significantly lower enzyme activities in the biocatalytic preparation of chiral sulfoxides (Chen et al., 2014; de Gonzalo et al., 2017; de Gonzalo and Franconetti, 2018). In most biocatalytic processes, the initial substrate concentration was usually less than 30 mM (Chen et al., 2014; Matsui et al., 2014; Wu et al., 2018; Zhang et al., 2018). The fact that a high concentration of unnatural substrates reduces enzyme activity makes substrate concentration be the bottleneck for biocatalytic preparation of chiral sulfoxides. In our previous *pm*MsrA whole-cell system, the catalytic efficiency also decreased greatly under the substrate concentration higher than 5 mM. Thus, we considered establishing a MsrA enzyme regeneration system to improve the initial substrate concentration (Peng et al., 2018). Based on the catalytic mechanism of MsrA, the 0.6-fold of *rac* substrate amount of dithiothreitol (DTT) was used as a reductant to regenerate the enzyme after catalysis. The (*R*)-sulfoxides were obtained with high enantioselectivity (*ee* > 99%, *E* > 200) under the substrate concentration up to 200 mM in 4 h. The substrate tolerance of this system was much better than most reported biocatalytic asymmetric oxidation strategies. Moreover, this system was also capable of preparing (*R*)-sulfoxides with substituents at the aromatic ring efficiently (Peng et al., 2018).

To screen MsrA enzymes with better catalytic properties, our group then tested the activities of several *pm*MsrA homologs from different species (Yang et al., 2019). Finally, a MsrA homolog from *P. alcaliphila* (*pa*MsrA) exhibiting remarkably excellent activity and enantioselectivity toward a series of aryl methyl/ethyl sulfoxides was obtained (Figures 1B,F). Chiral sulfoxides in the *R* configuration were prepared with approximately 50% yield and up to 99% *ee* through the *pa*MsrA catalyzed asymmetric reductive resolution. More importantly, the kinetic resolution was successfully accomplished with high enantioselectivity (*E* > 200) at initial substrate concentrations up to 320 mM (approximately 45 g/L) (Yang et al., 2019). The initial substrate concentration is 50 times higher than most of the reported native enzymes, representing a great improvement in the biocatalysis of chiral sulfoxides. Based on the above reports, the extremely high substrate tolerance and catalytic rate, as well as wide substrate scope and good operability of MsrA, implied an excellent industrial prospect. Compared with conventional asymmetric oxidation of sulfide, oxygen and high-cost cofactors, like NAD(P)H, are not needed in the MsrA catalytic kinetic reaction, which would greatly simplify the process and lower the cost for future industrial applications. Thus, these studies strongly support that the asymmetric reductive resolution of *rac*-sulfoxides using MsrA could be an ideal strategy for the green preparation of optically pure sulfoxides.

The main drawback of the Msr catalyzed system is that the yield of the enantiopure sulfoxide could only reach a maximum of 50%, which could be a great limitation at the industrial level. The re-use of the corresponding sulfide product would be necessary to improve the catalytic efficiency. Míšek’s group reported a chemoenzymatic dynamic deracemization system in 2018 (Nosek and Misek, 2018). By screening different chemical oxidants, an oxaziridine-type oxidant was used to combine whole-cell *E. coli* overexpressing MsrA to establish this biphasic system (Figures 1C,F). The mechanism is that once the enantioselective reduction of *rac*-sulfoxides occurred in the aqueous buffer, the corresponding sulfides were oxidized back to *rac*-sulfoxides in the decane phase, without inactivating the biocatalytic system. A series of *rac*-sulfoxides were converted into the *R* configuration with >99% *ee* and 55–93% yield. Although the biphasic system was complicated and the substrate tolerance of this system was not tested, this system provided an alternative technique and new thought for overcoming the yield bottleneck of MsrA (Nosek and Misek, 2018).

Although the MsrA has shown excellent substrate adaptability, the activities on the substrate with complicated structures are much lower (Yang et al., 2019). Particularly, a methyl or ethyl on the sulfinyl group is essential for the catalysis of MsrA (Yang et al., 2019). The limitation in substrate scope restricts the industrial application of MsrA. Therefore, modifications including directed evolution would be needed to improve the substrate compatibility. The development of a high-throughput screening assay to monitor the activity of MsrA is a priority. Recently, the Míšek group has developed a high-throughput fluorogenic assay for monitoring the activity of MsrA (Tarallo et al., 2020). A fluorogenic probe with propyl substituent called propyl GreenOx was used as a substrate to screen the random mutant library of MsrA, given the fact that a marked increase of the fluorescent signal would be observed if the mutant is active on this probe (Figure 1D). Finally, a mutant F52L with expanded substrate scope was obtained. Compared to the wild-type MsrA, which is only active on substrates with methyl/ethyl sulfoxide moiety, the mutant F52L was able to resolve substrates with propyl and butyl substituents (Figures 1D,F). The design and application of fluorogenic probes provide a good strategy for the high-throughput screening of MsrA mutants. The drawback of this method is that for every screening, specific fluorogenic probe substrates have to be designed and synthesized, while mutants active on one probe may not be active on other substrates. On the other hand, the consumption of cofactors, like NADPH or DTT, is also a way to monitor the activity of MsrA. For example, Wu et al. have reported a colorimetric microplate assay for testing MsrA activity (Wu et al., 2013). The method is based on the fact that MsrA catalyzes the reduction of sulfoxides with the consuming dithiothreitol (DTT), whose color can be produced by reacting with dithiobis-nitrobenzoic acid (DTNB). Thus, the corresponding absorbance change at 412 nm could be used as an indicator of MsrA’s activity. Compared with the fluorogenic assay mentioned above, these substrate-independent methods were also valuable as specific substrate probes are not needed and any substrate might be used for screening.

### Application of MsrB in Chiral Sulfoxides Preparation

In 2001, Grimaud et al. identified a new type of methionine sulfoxide reductase, named MsrB (Grimaud et al., 2001). The gene of MsrB exists in genomes of prokaryotes and eukaryotes but showed no sequence similarity with MsrA. The following reports revealed that MsrA and MsrB display opposite stereoselectivities in the reduction of Met-O. Further research showed that the MsrB catalyze protein-bound Met-O more efficiently than free Met-O (Olry et al., 2002). Afterward, various MsrBs active on the *R* enantiomer of Met-O were identified in multiple species, from prokaryotes to eukaryotes (Achilli et al., 2015). The study of the crystal structure of MsrA showed no resemblance to that of MsrB, but their active sites exhibited approximate mirror symmetry (Lowther et al., 2002). Structure analysis shows that the substrate is stabilized by hydrogen bonding interactions, including His103, and a water molecule, which itself interacts with His100, Asn119, and Thr26 (numbering based on the *E. coli* MsrB sequence) (Boschi-Muller and Branlant, 2014). Two antiparallel β-sheets surrounded by several helices form the core of the MsrB enzyme. Further comparison of the reduced and oxidized forms of MsrB reveals that this enzyme undergoes drastic conformational rearrangements of the two β-sheets upon oxidation, indicating that MsrB displays high structural flexibility (Drazic and Winter 2014). The activity of MsrB is usually lower than that of MsrA, for example, MsrB and MsrA enzymes in *E. coli* reduce free Met-O with turnover rates of 20 min^−1^ and 0.6 min^−1^, respectively (Grimaud et al., 2001). In some particular species, such as *Streptococcus pneumoniae*, *Neisseria gonorrhoeae*, and *Haemophilus influenza*, the MsrA and MsrB genes are fused and two enzymes exist as domains in a single fused protein (Kim et al., 2009).

Based on the fact that MsrB is specifically active on the *R* enantiomer of sulfoxides, the application of this kind of enzymes for preparation of (*S*)-sulfoxides through kinetic resolution was possible. Nosek and colleagues have investigated several MsrB homologs, but no candidate was obtained (Nosek and Misek, 2019). Considering that the Msr homologs widely existed in living species, our group tested the activities of a series of MsrB homologs to obtain enzymes for (*S*)-sulfoxides preparation. Finally, the first MsrB with good activity and enantioselectivity was discovered (Wen et al., 2020). The (*S*)-sulfoxides in 93–98% *ee* were obtained through the catalysis of recombinant MsrB from *Acidovorax* species (named *ak*MsrB), at initial substrate concentration up to 50 mM (Figures 1E,F). The establishment of the MsrB catalytic kinetic resolution system suggests that the MsrB could also be used to prepare chiral sulfoxides and provides a new efficient green strategy for the preparation of (*S*)-sulfoxides. Compared to MsrA, the MsrBs are less active and have higher substrate specificity (Wen et al., 2020), limiting their utilization. Screening enzymes with higher catalytic performance and further modification on this enzyme are essential before the industrial application of MsrB for the preparation of (*S*)-sulfoxides.

### DMSO Reductase

**FIGURE 2:**
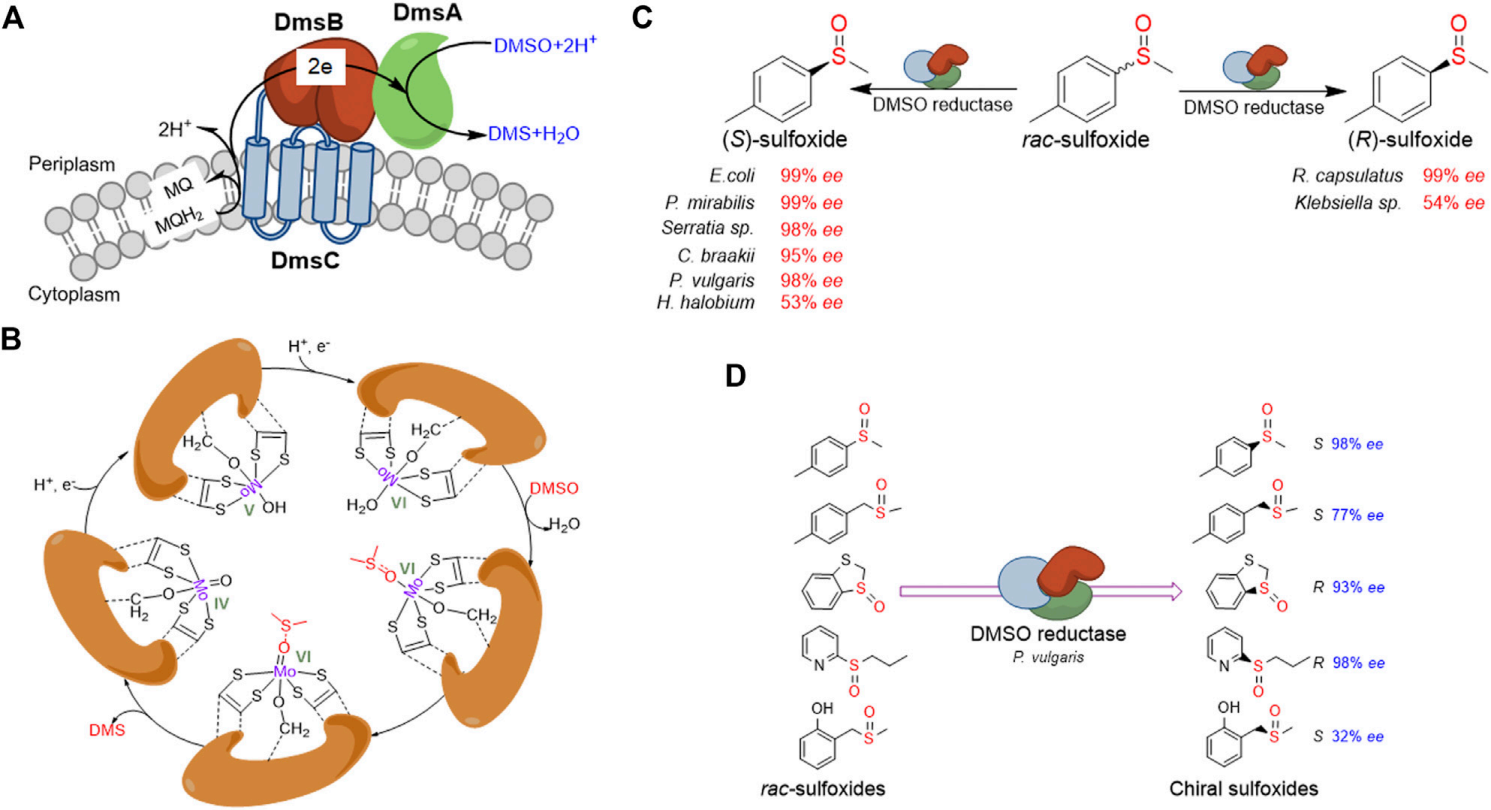
Overview of DMSO reductase and application in chiral sulfoxides preparation. **(A)** Typical constitution and electron transferring of DMSO reductase in *E. coli.* **(B)** Schematic presentation of the proposed catalytic mechanism for the catalytic cycle of DMSO reductase. **(C)** Representative presentation of DMSO reductases from different organisms shows opposite stereospecificity toward the same substrate. **(D)** Representative presentation of one DMSO reductase shows opposite stereospecificity toward different substrates.

DMSO reductase activity is widespread in microorganisms involved in anaerobic respiratory processes. In the absence of oxygen, respiration using alternative electron acceptors like DMSO instead of oxygen occurs in numerous microbes (McCrindle et al., 2005). Through using hydrogen, formate, or glycerol 3-phosphate as electron donor and DMSO as an electron acceptor, the DMSO reductase allows microbes to respire anaerobically. The function of DMSO reductase involves the oxidation of MQH_2_ to MQ, the transfer of two electrons from the membrane domain to the molybdenum active site, and the reduction of DMSO to DMS (Cheng and Weiner, 2007). In prokaryotes, the DMSO respiratory chains contain two main categories (McCrindle et al., 2005). The first DMSO reductase, typified by the *E. coli* DmsABC, belongs to a group of membrane-associated [Fe-S]-molybdoenzymes. This enzyme complex includes a molybdenum cofactor containing catalytic subunit (DmsA), an electron transfer subunit that contains groups of cysteine residues for assembly of [4Fe-4S] clusters (DmsB), and a membrane anchor and quinol binding subunit (DmsC) (Cheng and Weiner, 2007). The DmsA and DmsB are located outside the membrane and face the periplasm (McEwan et al., 2002; Cheng and Weiner, 2007) (Figure 2A). The second group of DMSO reductases includes a soluble periplasmic molybdenum-containing catalytic subunit and a membrane-associated c-type cytochrome that reduces the soluble molybdoenzyme (McEwan et al., 2002).

The catalytic subunit of DMSO reductase contains a molybdo-bis(molybdopterin guanine dinucleotide) (Mo-bisMGD) molecule at the active center. During the reduction of DMSO, the Mo atom cycles between the Mo (IV), Mo (V), and Mo (VI) states. Firstly, the Mo (VI) ion accepts one electron from the electron transfer subunit and one proton to form a hydroxideligated Mo (V) intermediate, followed by another one-electron/one-proton addition to yield the Mo (IV) form. The putative weakly bound water molecule is then displaced by DMSO, which is bound exclusively through the oxygen, with concomitantly weakening the S=O bond of DMSO. The electron-withdrawing ability of the *trans* dithiolene ligand to form the more localized dithiolate type, dithiolene, lastly drives the reformation of Mo (VI) and the release of product DMS (Figure 2B; Garton et al., 1997; Cheng and Weiner, 2007). Most DMSO reductases have broad substrate specificity, catalyzing the electron transfer from menaquinol to a wide variety of S-oxides and N-oxides (Cheng and Weiner, 2007). Recently, Makukhin et al. have reported that in stationary phase-stressed *E. coli* cells, the DMSO reductase exhibited the reduction activity of the (*R*)-isomer of methionine sulfoxide, instead of MsrB (Makukhin et al., 2019). This research revealed a new function of DMSO reductase contributing to the reparations of oxidative damage.

The application of DMSO reductase in the preparation of chiral sulfoxides through kinetic resolution started in the 1990s. Abo et al. have first described using DmsABC from *Rhodobacter sphaeroides* for the kinetic resolution of methyl phenyl sulfoxide and obtained (*R*)-sulfoxides in 35–42% yield and 50–99% *ee* (Abo et al., 1994; Abo et al., 1995). Afterward, researchers have investigated the kinetic resolution of *rac*-sulfoxides by DMSO reductases using a range of microorganisms (Hanlon et al., 1998; Boyd et al., 2004; Luckarift et al., 2004). A number of new bacterial isolates have been identified to be able to reduce a diverse range of *rac*-sulfoxides to yield the residual enantiomer with wide substrate specificity. Recently, Míšek and co-workers have reported the kinetic resolution of *rac*-sulfoxides using whole-cell *E. coli* DmsABC in aqueous buffer/decane biphasic conditions and showed high enantioselectivity (up to >99% *ee*) (Nosek and Misek, 2019). Unlike MsrA and MsrB, which are only active on one specifical enantiomer of *rac*-sulfoxides, the DMSO reductases from different organisms could show opposite stereospecificity (Hanlon et al., 1998; Boyd et al., 2004; Luckarift et al., 2004) (Figure 2C). For instance, DMSO reductase from *Rhodobacter capsulatus* catalyzes the reduction of (*S*)-sulfoxide, whereas DMSO reductases from *E. coli* and *Proteus* species reduce the (*S*)-sulfoxide. Moreover, the same DMSO reductase could also show opposite stereospecificity toward different substrates (Luckarift et al., 2004) (Figure 2D).

Based on the fact that DMSO reductase can accept electrons directly from artificial electron donors, studies on electrochemical DMSO reductase systems using an artificial electrode as an electron donor have been reported (Abo et al., 2000; Chen et al., 2015). For instance, Abo et al. have developed an electrochemical enzymatic system that consists of a glassy carbon electrode as the working electrode, methyl viologen as the mediator, and DMSO reductase as the catalyst. The (*R*)-sulfoxides with a variety of functional groups are obtained with high *ee* (>97%) through this system. In addition, the dynamic kinetic resolution using DMSO reductase combined with chemical oxidant has also been developed to improve the yield and *ee* of chiral sulfoxide products (Tudorache et al., 2012). Enantiopure sulfoxide is obtained by using *E. coli* cells to catalyze the reduction of (*R*)-sulfoxide to the sulfide, followed by using a heterogeneous Ta_2_O_5_-SiO_2_ catalyst for the oxidation of sulfide to the *rac*-sulfoxide. The sequential deracemization process applied for *rac*-sulfoxide is cyclically performed, which leads to 97.5% *ee* of (*S*)-sulfoxide and a 56% yield after three deracemization cycles.

Overall, the DMSO reductases have been proven to be a valid alternative method for synthesizing chiral sulfoxides. Compared to Msrs, the substrate scope of DMSO reductases is much wider. However, the substrate tolerance is much lower and the enantioselectivity is not good enough. Moreover, as a membrane-bounding protein with three subunits, which is kind of too complicated, DMSO reductases have mostly been used as a natural whole-cell form. The commonly used genetic engineering techniques are difficult to apply for improving the yield and activity of the enzyme. In addition, the anaerobic environment is usually needed to ensure the growth of the bacteria and upregulate the synthesis of the reductase. These shortages severely limit the development of DMSO reductases in the application of chiral sulfoxides preparation. Thus, more efficient ways to improve the enzymatic system will be needed in the future.

## Conclusions and Outlook

This mini-review mainly focuses on developing sulfoxide reductases and identifying their chemical and biological functions and applications in chiral sulfoxide preparation. The properties and prospects of these enzymes, especially MsrA, have been highlighted and discussed. The asymmetric reductive resolution of *rac*-sulfoxides catalyzed by these reductases provides a valid alternative way to prepare enantioenriched sulfoxides besides the conventional asymmetric oxidation strategy. Among these sulfoxide reductases, the MsrA exhibited extremely high substrate tolerance and catalytic rate, wide substrate scope, and low-cost and good operability in the reaction system, which implies very good prospects in industrial applications. However, the limitation in substrate structure (methyl or ethyl on the sulfinyl group was essential) blocked its application. Future efforts in protein engineering like directed evolution for the improvement of substrate adaptability are still needed. Compared to MsrA, the MsrB exhibited relatively poor catalytic properties in the aspects of catalytic efficiency, substrate tolerance, and substrate scope. Therefore, screening homologs with better catalytic properties are the primary issues to be resolved. Compared to Msrs, the DMSO reductase displayed better substrate adaptability but worse substrate tolerance and catalytic rate. Moreover, the complexity in composition and structure and the difficulties in protein expression limited the improvement of DMSO reductase through biological techniques. Efforts in developing novel catalytic systems would probably offer new ways to synthesize enantioenriched sulfoxides using this enzyme. At last, although these enzymes provide excellent alternatives for the preparation of chiral sulfoxides, the natural defect of 50% maximum theoretical yield for kinetic resolution cannot be ignored. Developing dynamic reduction-oxidation enzymatic cascade reaction might be a solution to overcoming this drawback in the future.
